# The Official Story of the Law

**DOI:** 10.1093/ojls/gqac028

**Published:** 2022-10-29

**Authors:** William Baude, Stephen E Sachs

**Affiliations:** Professor of Law, University of Chicago Law School; Antonin Scalia Professor of Law, Harvard Law School

**Keywords:** positivism, Hart, officials, acceptance, rule of recognition, recognitional community

## Abstract

A legal system’s ‘official story’ is its shared account of the law’s structure and sources, which members of its legal community publicly advance and defend. In some societies, however, officials pay lip service to this shared account, while privately adhering to their own unofficial story instead. If the officials enforce some novel legal code while claiming fidelity to older doctrines, then which set of rules—if either—is the law? We defend the legal relevance of the official story, on largely Hartian grounds. Hart saw legal rules as determined by social rules accepted by a particular community. We argue that this acceptance requires no genuine normative commitment; agreement or compliance with the rules might even be feigned. And this community need not be limited to an official class, but includes all who jointly accept the rules. Having rejected these artificial limits, one can take the official story at its word.

## 1. Introduction

In some societies, officials use conventional legal reasoning as a fig leaf. They pay lip service to conventional rules in public, while privately assessing and guiding their conduct by very different standards. Soviet judges, for example, famously followed a hidden chain of command: they decided cases based on telephoned instructions from party officials, and they made up the public explanations later.^1^ Modern authoritarian states still promulgate ‘sham laws’ they have no intention of obeying, or impose ‘hidden laws’ they would never admit to enforcing; their private rules of decision depart drastically from their public legal reasoning.^2^

These departures pose an important and understudied issue of legal theory. What is the law—if anything is—when officials acknowledge one set of rules in public and another in private? To a legal positivist, ‘what counts as law in any society is fundamentally a matter of social fact’.^3^ Orthodox modes of legal justification are social facts, but so are the unorthodox norms of legal officials, and sometimes the two disagree. Positivists need an account of what this disagreement means for the law.

The need is particularly serious for positivists in the tradition of HLA Hart. Hart argued that legal rules are grounded in social rules, accepted by the right people in the right way as the right means of determining the law.^4^ A social rule of recognition is reflected in what one might call the legal system’s *official story*: its shared account of the law’s structure and sources, which members of its legal community publicly advance and defend.^5^ The trouble is what to do with an *unofficial story*: a ‘systematic practice of officials, guided by general rules, which is inconsistent with the “official story” of the law that officials sell to the public’.^6^ If officials are guided by one rule of recognition in their public arguments and another in their private decisions, then which rule, if either, determines the society’s law?

The question has practical as well as theoretical importance. Departures from the official story are hardly limited to dark corners of lawless regimes. Consider the United States, where even critics of originalism describe it as ‘the legal profession’s orthodox mode of justification’.^7^ Officials across the political spectrum invoke the original Constitution’s authority to ground their own;^8^ they claim that ‘we are all originalists’, committed to applying the ‘specific rules’ or ‘broad principles’ that the founders ‘laid down’.^9^ Overt official rejections of the founding’s relevance yield the sort of ‘criticism’ and ‘demands for conformity’ that positivists might expect.^10^ Yet US officials often reach decisions that seem inconsistent with these commitments: say, ascribing to Congress broad economic powers to regulate wheat grown and eaten at home,^11^ something ‘difficult if not impossible to justify on originalist grounds’.^12^

How should one explain this divergence? Perhaps originalist commitments are not really so widespread.^13^ Perhaps the officials disagree about what originalism requires; or perhaps they suffer from motivated reasoning,^14^ and so they apply the ‘broad principles’ of the founding in unfortunate or mistaken ways.

Yet some critics advance another, more troubling explanation: the officials simply employ another rule of recognition, about which they have ‘not been fully candid’ with the public.^15^ Whatever official story they might ‘publicly avow’ in speeches or confirmation hearings,^16^ in chambers they adhere to an unofficial story instead: to ‘an evolutionary common law system’,^17^ to a Supreme Court acting as ‘super-legislature’^18^ or to ordinary policy preferences accompanied by ‘ritual obeisance to the founders’.^19^ These officials might not *admit* ‘that some other source of authority trumps original meanings’^20^—they would never reject such meanings ‘*overtly*’^21^—but neither do they follow those meanings ‘wherever they might lead’.^22^ Rather, they ‘employ an originalist rhetoric that persuades citizens, who do not quite acknowledge that a number of decisions they like fail under originalist standards’.^23^ Because this official rhetoric is merely rhetoric (the argument goes), and because non-official views do not matter, the unofficial story determines the law.

We doubt that this conspiratorial account is true, as a descriptive matter; we do not take non-originalists to be acting in bad faith.^24^ But we also think it mistaken as a matter of theory. To frame things in stark terms, consider the following scenario:

*Illuminati*: The American legal system appears to most observers entirely ordinary. Judges and other officials explain their decisions by citing its Constitution, statutes, treaties, common law, etc. But a few canny professors come to realise that the officials are all part of an Illuminati conspiracy, choosing outcomes according to secret Illuminati directives while purporting to justify them under the Constitution and other sources.^25^

Should we say that the secret Illuminati directives *are* the law of the United States, defining the legal rights and obligations of its citizens? Or—as seems plain to us—have the officials formed a conspiracy to *subvert* American law?

To defend the latter intuition, we offer two refinements on the Hartian approach, respecting issues on which Hart’s account seems unclear: what it means to ‘accept’ a social rule, and whose acceptance matters in determining the law. If ‘ultimate rules of recognition are grounded exclusively in *genuine* acceptance by *legal officials*’, as Mikołaj Barczentewicz suggests,^26^ then the unofficial story is indeed the one that counts: it is the only story genuinely accepted by the only people who matter. Public thought and talk about the law’s structure and sources would then be deeply mistaken and in need of revision, to better conform to the officials’ private rules. By contrast, if acceptance need not be genuine in this sense (if dishonest or superficial commitments are enough), and if the recognitional community includes other members of the legal community as well, then the official story may win out: it is the only story at-least-thinly accepted across the board. In that case, the legal community’s existing account of the law’s structure and sources could be taken at its word.

There is good reason to favour the latter view. Many of Hart’s insights arise from law’s similarities to other artificial normative systems—which range, as Mitchell Berman points out, from informal systems such as fashion or etiquette to highly formalised systems such as sports and games.^27^ None of these *other* systems rests on the genuine commitments of some narrow subclass of participants. One may accept the rules of baseball in a superficial way, as rules of the game, without endorsing them as claims about ‘what its players really ought to do’^28^—indeed, without any genuine intention to comply with these rules, in the hope that one’s cheating will go undetected. And such rules are the common property not just of umpires and officials, but of players and fans as well.

The same is true for law. One might accept, in the same superficial way, a social rule that identifies legal rules. Judges who secretly subvert this social rule still treat the rule *as if* it were binding; they co-operate in maintaining the rule’s existence by displaying the relevant normative attitudes to others. And the relevant community by which the rule is accepted is not some predefined set of officials, but the legal community writ large: the community of those who jointly accept the rule, using a shared story as an ‘essentially common or public’ mode of justification.^29^ The hidden rules of officials might still matter in practice, just as it matters to litigants whether a judge has been bribed. Yet even a standardised schedule of bribes ought to be distinguished from the legal system behind whose rules it hides.

The content of the law thus depends more on abstract features of legal rhetoric and justification than on the norms that legal officials enforce in practice. This conclusion might seem surprising, given Hart’s insistence that legal systems be efficacious on the ground.^30^ Surely Congress may regulate even unto the last grain of home-grown wheat, if courts and prosecutors reliably obey its commands? Yet, as Hart emphasised, legal rules play an internal, evaluative role as well as an external, descriptive one; they rest on practice without reducing to external observation or prediction.^31^ Hart thereby captured something rich about the social understanding of law, something that distinguishes law from the unspoken conventions of an official class. To explain it, we ought to pay close attention to the official story—whether in academic hypotheticals or in our own familiar world.

## 2. Acceptance

The *Illuminati* hypothetical envisions not just widespread law breaking or government corruption, but also a broad commitment by officials to coherent rules, giving the official story a real competitor: an unofficial story that makes the official one seem false or feigned. To the Illuminati, the public legal rules are like the ‘rules’ of professional wrestling.^32^ The officials’ real social rule is to pretend to follow them, not to do so in fact.

This division leaves the state of the law unclear. To some scholars, accepting a social rule means holding a genuine belief in its value: if a group does not ‘genuinely believe [a] standard to be binding’, then it is not their standard of behaviour.^33^ While the Illuminati officials may mouth the words of the public legal rules, Barczentewicz argues, they are ‘more fundamentally committed’ to their private Illuminati rules than to these ‘abstract declarations of principle’.^34^ The officials thoroughly accept the Illuminati rules, which the citizens end up obeying. That suffices to ground a single legal system, in which the true law is Illuminati law.^35^

Yet the depth of official commitments might not matter. When it comes to other normative systems, such as games, fashion or etiquette, we do not expect anyone to share genuine beliefs that certain poker hands, outfits or behaviours are truly superior or right. We just expect participants to display, by their public conduct, a certain attitude towards the rules. Consider those who secretly hate the game of poker, who believe its rules to be unfair and who only play to keep their grudging promises to friends; such players still apply, maintain and contribute to the existence of a system of norms. Importantly, so do those who secretly *cheat* at poker, for the rules are what tell them *how* to cheat, and only by invoking the rules with other players can their cheating be of use. (An ace up the sleeve presupposes that aces are worth having.)

Expecting legal officials to share a genuine commitment to their legal rules is expecting too much. The only commitment needed to accept a rule is the rather superficial one already involved in adopting the internal point of view: the commitment to use it as an ‘essentially common’ standard of behaviour. This kind of acceptance might be thought illusory or dishonest, but for law’s purposes, it is genuine enough.

### A. Acceptance and Belief

To see what sorts of commitment a Hartian social rule requires, we might ask why social rules figure in Hart’s account in the first place. In our view, the best explanation is as follows. Social rules illustrate important features of social life that purely external accounts might miss. People ‘*use* the rules as standards for the appraisal of their own and others’ behaviour’,^36^ standards which they ‘voluntarily co-operate in maintaining’.^37^ Thus, we can speak of chess as having a system of rules, rather than of chess players merely having ‘similar habits of moving the Queen in the same way’,^38^ partly because the players share a ‘characteristic vocabulary’.^39^ They use words like ‘“ought”, “must”, and “should”, “right” and “wrong”’,^40^ much the way referees might use ‘the expression “Out” or “Goal”’: as ‘the language of one assessing a situation by reference to rules which he in common with others acknowledges as appropriate for this purpose’.^41^

Hart differentiates legal rules from external accounts of official behaviour in roughly the same way. From the external perspective, for example, the tax collector and the local gunman look the same; each demands money on pain of sanction and is habitually obeyed.^42^ Yet, unlike the gunman, the tax collector plays a role that is ultimately defined by social rules, accepted from the internal point of view.^43^ Legal norms are more than predictions of what legal institutions or individual officials will do: they are standards of behaviour identified by a system of social rules—including ‘a complex rule of recognition’ containing a ‘hierarchical ordering of distinct criteria’, as revealed in ‘the general practice of identifying the rules by such criteria’.^44^ Those who accept such social rules co-operate in maintaining them as ‘common standards of legal validity’, not things that ‘each judge merely obeys for his part only’.^45^ So, to the extent that members of a legal community share an official story—that is, to the extent that they offer a shared account of their law’s structure and sources, publicly advancing and defending that account as a justification for legal claims—this official story serves to illustrate whatever rule of recognition generally obtains.

None of this seems to depend on the depth of anyone’s commitments. We can differentiate ‘the adult chess-player’s move from the action of the baby who merely pushed the piece into the right place’^46^ without assuming that the chess player enjoys playing chess, normatively endorses its rules or would hesitate to move another player’s pieces if no one were looking. Hart’s view of acceptance is not fully clear,^47^ but it seems that he is after—or ought to be after—something weaker than actual normative agreement. Those who accept a social rule, in this thin sense, need not agree that it accurately specifies what members of the group really ought to do. But they do take the rule as the sort of thing appropriate for invoking the word ‘ought’—as ‘exhortative and injunctive’,^48^ in Benjamin Zipursky’s phrase, ‘purport[ing] to direct individuals to act or not to act in a certain manner’.^49^ And while they may despise the rule, or secretly envy those who break it, they nonetheless co-operate in maintaining it—such as by expressing criticism of violators, acknowledging such criticism by others and acting in ways that confirm rather than undermine its general adherence.

Acceptance of this kind is about intelligibility, not motivation or belief.^50^ The relevant attitude finds its ‘characteristic expression in the normative terminology of “ought”, “must”, and “should”, “right” and “wrong”’^51^—terms one might *use* in regard to social rules without actually *believing* that the rules establish what is right and wrong.^52^ Rules may be accepted in specific contexts and for specific reasons; to borrow an example from Adam Perry, an employee’s acceptance of a rule that ‘the customer is always right’ is usually (and properly) sloughed off as soon as the customer departs.^53^ Perry emphasises how the acceptance of rules can be belief-independent, with individuals acting ‘as they would act were they to believe the normative proposition that is the rule’s content’, regardless of whether they actually do.^54^

Indeed, Hart specifically distinguishes acceptance of a rule from such ‘feelings of compulsion’.^55^ He recognises that ‘those who do accept the system voluntarily’ need not ‘conceive of themselves as morally bound to do so’, and he suggests that they may base ‘their allegiance … on many different considerations’, such as ‘long-term interest’ or ‘the mere wish to do as others do’.^56^ As Scott Shapiro colourfully puts it, ‘Judges might apply the law simply to pick up their paychecks’.^57^ For these common norms to be intelligible, their participants merely have to understand them as common norms, which for some practical purpose they choose to participate in expressing.

This understanding casts a different light on Hart’s ‘critical reflective attitude’—an attitude that ‘display[s] itself in criticism (including self-criticism), demands for conformity, and in acknowledgements that such criticism and demands are justified’.^58^ For behaviour to be looked upon ‘as a general standard to be followed by the group as a whole’,^59^ it is crucial that criticism and demands for conformity be regularly *expressed* and *acknowledged*, but not that such criticism or acknowledgements be heartfelt. The rules of poker could survive as intelligible features of social life even if each player were secretly happy to cheat undetected and felt no real guilt when caught. Yet if open and notorious violations no longer led to acknowledged criticism, or if poker players no longer kept up the appearance of complying with the rules, then the game would no longer be intelligible; it would no longer involve a system of rules that participants ‘voluntarily co-operate in maintaining’.^60^

The same goes for Hart’s suggestion that, if a rule of recognition ‘is to exist at all’, it ‘must be regarded from the internal point of view as a public, common standard of correct judicial decision’.^61^ For courts to be ‘critically concerned with … lapses from standards, which are essentially common or public’,^62^ the judges do not have to feel any subjective sense of guilt when their lapses are discovered; but they do have to maintain the ‘essentially common or public’ nature of the standards themselves. The depth of the officials’ actual commitments might affect ‘the efficiency or health of the legal system’, but their heartfelt devotion is not a condition of its intelligibility, ‘of our ability to speak of the existence of a single legal system’.^63^ Intelligibility depends on whether the common standards, written or unwritten, are deployed *as common standards*, not whether they reflect the true motives of individual behaviour.

Even in their attempts to subvert it, then, Illuminati officials nonetheless accept the official story. By adhering in all their public conduct to the outward forms of American law, and by expressing and acknowledging criticism for any noticed deviations from those standards, they adopt precisely the same attitude towards the law’s structure and sources that is necessary to accept a shared rule of recognition—and, indeed, a shared official story.

### B. Genuine, Dishonest and Fictional Acceptance

One might criticise this thin account of acceptance as too thin. A judge who applies the law to pick up a paycheck might nonetheless apply the law faithfully.^64^ But what of the judge who merely *pretends* to apply the law, while consistently rendering decisions contrary to her best understanding of what that law requires? Barczentewicz, who accepts most of Perry’s account, distinguishes ‘genuine’ acceptance from acceptance that is merely ‘pretended’ or feigned.^65^ To *genuinely* accept a rule is to have certain dispositions, such as to ‘act in accordance with the rule’, to form ‘a reactive attitude of blame towards rule breakers’ or to adopt ‘normative expectations that others (rule addressees) conform’.^66^ By contrast, he argues, Illuminati judges are not *genuinely* disposed to obey American law. They do not form reactive attitudes of blame towards those who violate it; at best, they ‘have dispositions outwardly to pretend they blame’.^67^ Like actors who manifest false attitudes on stage, or like a network of spies ‘pretending to accept the rules of groups they infiltrate’, the Illuminati officials lack ‘the appropriate dispositions’ for those who genuinely accept a social rule.^68^

We agree that stage actors need not accept the norms they act out on stage. Thus, Perry’s condition for participating in the internal aspect of a rule—that agents have a belief-independent ‘attitude that leads them to act as they would act were they to believe the normative proposition that is the rule’s content’—seems too weak.^69^

At the same time, Barczentewicz’s requirement of genuine approval or blame seems too strong. It overlooks a crucial distinction between *fictional* expressions, such as those of a stage actor, and *dishonest* expressions, such as those of a cheater aiming to violate the rules undetected. Actors on stage can perform a scene depicting a poker game without actually playing poker; they might be engaging in a fiction of playing poker, and so (for the moment) they might only fictionally accept poker’s rules. But the dishonest player who draws an ace from his sleeve *is* playing poker, albeit dishonestly. Unlike an actor who merely *recites* ‘language … assessing a situation by reference to rules’,^70^ the dishonest player *uses* such language to *make* such assessments. He acts in ways intended to co-operatively maintain the rules (indeed, he hopes to claim undeserved winnings under them), and he takes those rules as relevant grounds for expressing or acknowledging criticism.^71^ His statements of criticism or acknowledgement are not ‘detached’ or ‘hermeneutic’ reflections on the commitments of others,^72^ but straightforward expressions of the relevant attitudes, made with all the intentions necessary for them to have their ordinary speaker’s meanings: that his hearers believe his expressions, that they recognise his intention to produce such beliefs and so on.^73^ By contrast, if the dishonest player took his audience to be in on the game, then he really would be play-acting, and his utterances would be fiction or sarcasm rather than expressions of the relevant attitudes.^74^

Dishonest acceptance may not be ‘genuine’ in the sense of being heartfelt, but it is genuinely a form of acceptance, genuine enough to do all the work Hart’s theory requires. For, while a social rule, to exist, requires *genuine expressions* of certain attitudes (expressions that are meant to be believed), it does not require expressions of *genuine attitudes* (attitudes that the speaker truly holds). Consider Perry’s example of a college student who accepts new rules of conduct ‘to fit in with the members of the group’^75^—each of whom might be doing the same thing. In one study, college students who were ‘privately less comfortable with excessive drinking than they believed others to be’ still thought themselves ‘expected to celebrate intoxication … as a test of group loyalty’ and to direct ‘social approval toward those who drink and disapproval toward those who do not’.^76^ Perhaps every student feels this way: no one enjoys the nightly drinking rituals, but each feels bound to participate, to pretend to enjoy them and to criticise (with apparent feeling) those who show insufficient enthusiasm. If the students’ true feelings became common knowledge, the social rule would likely vanish.^77^ Yet a well-informed sociologist would properly conclude that there *is* a social rule among the group: indeed, perhaps a very powerful one, which the students still co-operate in maintaining through their rule-conscious behaviour. (And this remains true even if no student genuinely complies with the rule, say, by dumping their drinks in the sink while no one is looking.^78^)

One might be tempted to redefine the social rule as follows. Perhaps it requires the students to *feign* heavy drinking, with sanctions applied to those who feign it poorly. The group’s pro-drinking ‘rule’ would then be more like a ‘rule’ of professional wrestling—and to criticise professional wrestlers for their non-compliance with official rules, as opposed to their acting skills, is an exercise in missing the point. But redefining the rule this way misdescribes the group’s social life. It is crucial to the culture of professional wrestling that the wrestlers’ pretence is common knowledge; it may be crucial to the student group’s drinking norms that the students’ true beliefs and actions are not. The students do not sigh with relief each night because they have successfully *complied* with a pro-feigning rule, but because no one has discovered their *violations* of a pro-drinking rule. The standard under which they criticise or acknowledge criticism, the standard that is ‘essentially common’, is a standard that requires drinking, not keeping up appearances. (Their show of compliance is dictated by prudence and a desire to remain in the group, not by any social rule.)

Alternatively, one might claim that the group has no social rules about drinking at all: the students mistakenly *believe* that it does. Certainly, the students are wrong about many things. Yet they are *not* wrong to understand certain actions, as described by rules, to be socially required of them by other students in the group. What more could a social rule need? If those living under a tyranny all wish they could escape the regime—the unhappy tyrant among them—would this prove that their expressions of fanatical enthusiasm are not demanded by social rules? What good is our concept of a social rule if it leaves such things out?^79^ The analytical benefit of social rules is that they make the life of a group more intelligible, and they do the same work here—whether their heartfelt endorsement is shared by a majority of group members, by only a few or by none at all.

In any case, if one absolutely insisted, one could always apply some other label to dishonestly accepted social rules—say, calling them social ‘schmules’ instead. But then one would have no reason to deny that a legal system might rest on a *schmule* of recognition. Such a legal system might be unstable in practice, but it would be just as intelligible as those we know best.

The Illuminati thus accept the official story in whatever sense is necessary to establish an intelligible social rule of recognition. They see it as important, for reasons of their own, to adopt (dishonest) attitudes of respect towards the orthodox structures and sources of law, and to engage in (dishonest) expressions criticising those who depart from these sources or acknowledging such criticism. Their attachment to the official story may rest on subterfuge, but for law’s purposes, it is real enough.

## 3. Officials

However thin or thick one’s account of acceptance, there remains the question of *who* must do the accepting. The thin account of acceptance described above could be vulnerable to another objection, namely that it yields too many legal systems at once. The Illuminati officials are playing a double game: their expressions of criticism and acknowledgement are intended to convey one meaning to the public and another to their fellow conspirators. Within their own group, the pretence *is* common knowledge, and the rules of the broader legal system do resemble the rules of professional wrestling; the ‘essentially common’ standard among the Illuminati is to feign adherence to those rules, not to adhere. So, if the Illuminati accept both stories, official and unofficial, and if theirs is the relevant community for determining American law, then we seem to have two social rules and two bodies of American law rather than one. Indeed, between the two, the unofficial system may deserve pride of place, for the officials are ‘more fundamentally committed’ to their own private rules.^80^

Certainly, there are two distinct social rules in place: one among the Illuminati officials, one they share with the public at large. But why should the exclusive official class determine the law for everyone else? Scholars in the Hartian tradition have often suggested as much, but we see no good reason for it. Other artificial normative systems have officials but are not defined by them; the same could be true of law.

The social rules that determine the law, like other social rules involved in other artificial normative systems, more plausibly rest on the beliefs and practices of those who jointly partake in them. The rules of chess are determined by an amorphous ‘community of chess players’,^81^ those who recognise each other as playing a shared game. The rules of law are determined by a similar community of law users, who recognise each other as participating in a shared legal system. These individual participants may occasionally be ignorant of or mistaken about their shared rules, but they may also outsource their conclusions to narrower networks of knowledge and expertise, adopting the recognised experts’ views as their own. If so, the content of the law would reflect not the private commitments of officials, but an official story that unofficial participants may share.

### A. Officials and Social Rules

Hart is usually read as treating ‘Officials, not citizens’, as the recognitional community—the group whose attitudes and practices determine the law.^82^ The standard argument is as follows. In complex legal systems, primary rules of behaviour are identified by secondary rules rather than known directly to the public; ordinary people might have no idea how to identify the law.^83^ (As Leslie Green points out, many Californians are not ‘aware that there *is* a state constitution, so the sense in which the community “accepts” it is pretty attenuated’.^84^) Hart’s account thus looks to officials for acceptance, and to the general populace only for obedience.^85^ A society’s rules of recognition must ‘be effectively accepted as common public standards of official behaviour by its officials’, who engage with the rules *as* rules: approaching them from the internal point of view, ‘apprais[ing] critically their own and each other’s deviations as lapses’ and so on.^86^ The social rules of the official class identify legal rules, which count as the law in force if they are ‘generally obeyed’.^87^ On this view, Matt Adler suggests, the society’s ‘rule of recognition supervenes on official actions, beliefs, judgments, etc., alone’; the beliefs of private persons ‘have no role in determining its content’.^88^

In many societies, of course, ordinary people do engage directly with legal rules. Yet Hart denies that such engagement is necessary. Subjects alienated from their own government—say, in a society colonised by a foreign power^89^—might just as well regard the rules from the external view only: as if the officials were unpleasant robots, with mostly predictable behaviour (‘they’ll ticket me if I drive this fast’) but without normative commitments or indeed any inner life. That picture might be just as good, for ordinary purposes, as a coherent understanding of the traffic code and its place in the legal system.^90^ ‘In an extreme case,’ Hart suggests, ‘the internal point of view with its characteristic normative use of legal language (“This is a valid rule”) might be confined to the official world.’^91^ Such a society ‘might be deplorably sheeplike; the sheep might end in the slaughter-house. But there is little reason for … denying it the title of a legal system.’^92^

We are willing to assume that legal systems can survive without deep engagement by the populace. But that assumption does not entail that such engagement, *where it exists*, is irrelevant to a legal system’s content—much less that the recognitional community is always and everywhere restricted to officials. Patients in a hospital may be able to survive on intravenous fluids, but one should not conclude from this that solid food is irrelevant to the quality of one’s diet. Likewise, a rule of recognition can survive in the face of sheeplike incomprehension by ordinary people, but this does not render irrelevant the views of those non-officials who *do* accept it. Officials may keep the law on life support, but the content of a normal and flourishing legal system rests on more than official beliefs.

Indeed, the problems Hart sees as arising from secondary rules, and to which official recognition might be offered as the solution, can arise under any set of rules—with officials or without. In a simple society governed solely by primary rules, Hart concedes that some people might reject the rules or be alienated from them; a primary social rule (say, ‘no hats in church’) might exist despite some ‘who not only break the rule but refuse to look upon it as a standard either for themselves or others’.^93^ Yet we do not need an official class to bring these recusants to heel. So long as an influential subgroup *does* accept the rule (say, the handful of busybodies in the front pews), this group might successfully impose the rule on everyone else. Each ordinary member might comply ‘for his part only’, simply to avoid a nasty look—which, for Hart, is enough to create a social rule.^94^ Thus, Michael Wilkinson argues,

the difficulty in drawing the line between a simple society with a *powerful* minority and the complex legal world with its *official* minority suggests that the difference between simple and complex should not be relied upon to do too much work.^95^

### B. The Analogy to Games

In a sheeplike society, the recognitional community is plausibly limited to officials: they are the only people who regard the relevant rules from the internal point of view. In ordinary societies, ordinary people can do this too, so any special recognitional status for officials would have to rest on some *other* special property that officials have. Yet when we consider other artificial normative systems, no similar property reveals itself—making it very unlikely that any such property exists for law.

Consider the following scenario:

*Illuminati Baseball*: A nation’s baseball games, from professional leagues to kids’ games, appear to most observers entirely ordinary. Umpires and other officials explain their decisions by citing baseball’s traditional rules. But a few canny sportswriters come to realise that the officials are all part of an Illuminati conspiracy, making calls according to secret Illuminati directives while purporting to justify them under the traditional rules.

Should we say that the secret Illuminati directives *are* the rules of baseball? Or—as seems plain to us—have the officials formed a conspiracy to *subvert* baseball’s rules?

The strangeness of the *Illuminati Baseball* hypothetical, and of its suggestion that the players are mistaken about which game they are playing, weakens the case for Illuminati law. Why are umpires the only ones who matter? The players still play baseball in the ordinary way, regarding its rules from the internal point of view, and so on. Surely their beliefs and practices are relevant to determining the content of their game. So who is the legal theorist to contradict them, and to insist on the umpires’ views instead? An Illuminati baseball game may well be *unstable*; if the ‘aberrations are frequent, or if the scorer repudiates the scoring rule’, Hart suggests, there must ‘come a point when either the players no longer accept the scorer’s aberrant rulings or, if they do, the game has changed’.^96^ But when that point comes is an empirical issue, not a conceptual one. Until then, the players keep on playing the game as they understand it, grumbling about what seems to them like the occasional bad call. That there remains some period in which the game has *not* changed, though the umpires’ beliefs and practices already have, shows that the umpires’ beliefs and practices do not exclusively determine the rules.

In real-life baseball games, in which officials and players share the same rules, ‘the declarations of officials (umpire or scorer) have a special authoritative status attributed to them by other rules’.^97^ Even so, Hart writes, there is always ‘the possibility of a conflict between these authoritative applications of a rule and the general understanding of what the rule plainly requires’.^98^ This ‘general understanding’ is ‘general’ precisely because it is broadly shared, determined by the beliefs and practices of a broader group than the officials alone. Indeed, in responding to the rule-sceptics, Hart argues at great length that adding authoritative officials to an existing game changes nothing fundamental.^99^ Suppose that a group of friends are playing a pickup baseball game, accepting and applying its rules from the internal point of view. Their choosing another friend to serve as umpire does not change the scoring rule (much less reduce the game to one of ‘umpire’s discretion’); it merely adds one more rule to the list, conferring on the new umpire the in-game authority to adjudicate disputes about the rules. By the same token, adding officials to a set of social rules should not have the effect of drastically changing the other rules’ content or existence conditions, which remain as they were before.

This analogy to games severely undermines common arguments for restricting the recognitional community to legal officials. Kenneth Himma, for example, argues that because citizens do not ‘make, change, or adjudicate law’, they are not ‘*participants* in conventional legal practice’ and thus do not ‘directly figure, as a conceptual matter, into the existence conditions for a rule of recognition’.^100^ Barczentewicz likewise contends that private citizens have no direct authority over the law’s content; they can lobby, vote or protest, but their actions only affect the law as ‘mediated by officials’: ‘Whatever non-officials can do does not count as direct influence over the law’.^101^

Set aside, for the moment, factual objections based on jury service, referenda, citizen’s arrests or private powers to contract or convey. The deeper problem with this argument is that it mistakes a social function for a legal power. Those whose beliefs and practices *determine* a social rule of recognition need not be the same people on whom are *conferred* legislative or judicial authority by laws which the rule happens to validate.^102^ In the pickup game, the umpire has a unique adjudicative authority to make binding determinations of the score, precisely because the players recognise a rule conferring on the umpire just this kind of authority. That is the reason—the only reason—why the players’ determinations do not ‘count as direct influence’ on their own. The players can still assess the score for themselves, can still argue with the umpire and can still disregard the umpire’s authority and resume playing without one. The authority-conferring rule is not pulled up by its bootstraps from the umpire’s beliefs and practices; it rests on the players’ beliefs and practices too.

The same goes for the other rules. Three outs do not end an inning merely because umpires tend to think so. One could imagine a game in which the authority-conferring rule were the only rule the players recognised, with all else left up to umpires and officials; or a sheeplike baseball game in which the players, unaware of any rules, habitually obeyed the umpire and made guesses about which actions might be rewarded in the score. But most people play baseball, not ‘umpire’s discretion’.^103^

Once we separate the social function of recognition from the legal power of authoritative resolution, it becomes hard to see why any particular category of ‘officials’ must serve for both. Consider Neil MacCormick’s view that a social rule of recognition ‘states the duty of judges’ and must be accepted ‘by at least the judges and other superior officials exercising powers within the system’.^104^ When applied to games, this claim breaks down: baseball’s ‘rule of recognition’, whatever it might be, does not state the duty of umpires only, and whatever criteria yield the rule that there are three outs in an inning are criteria for umpires and players both. In the same way, rules of recognition may ‘state[] the duty of judges’ only insofar as they guide anyone seeking to identify the system’s rules.^105^ True, if a society’s judges and superior officials have all gone rogue—if they fail to accept, in the thin sense described above, roughly the same criteria of validity as others do—the society’s legal practices may seem too fractured to be intelligible. But the same is true in baseball if the umpires have all gone rogue, in which case one will likely end up either with different umpires or with a different game. To take any further Hart’s description of a rule of recognition as a ‘standard of correct judicial decision’^106^ would greatly overemphasise the importance of judges and courts. (Whether solicitors belong in the recognitional community does not seem to turn on whether they are ‘officers of the court’ or mere licensed professionals; their beliefs and practices seem equally relevant either way.)

To complete the analogy to games, private citizens in modern societies plainly occupy the role of players in the legal system, not just fans or spectators. As Hart notes, law’s ‘principal functions … are not to be seen in private litigation or prosecutions’, but in ‘the diverse ways in which the law is used to control, to guide, and to plan life out of court’.^107^ These include many rules ‘conferring private powers’, such as rules of contract or property, which ‘make[] of the private citizen’ a ‘private legislator’ and must ‘be looked at from the point of view of those who exercise them’.^108^ Indeed, it is hard to imagine anyone buying a house (obtaining financing, comparing offers and counteroffers, going to closing and so on) without once accepting the law in the sense described above—without once treating the law as a source of common standards of conduct or without once resorting to ‘characteristic vocabulary’ such as ‘it is the law that …’, ‘ought’ or ‘should’. As Hart points out, one finds these phrases ‘on the lips not only of judges, but of ordinary men living under a legal system’, who routinely assess new situations ‘by reference to rules which [they] in common with others acknowledge[] as appropriate for this purpose’.^109^ Perhaps that is why, notwithstanding his claims about officials, Hart repeatedly includes private citizens among those who shape the rule of recognition—‘a complex, but normally concordant, practice of the courts, officials, *and private persons* in identifying the law by reference to certain criteria’.^110^

### C. Recognition and Shared Acceptance

If the recognitional community is broader than the class of officials, then how broad is it? The class of ‘citizens’, for example, might turn out to be too broad, or perhaps not broad enough. As Green notes, ‘“The people of California” is an abstraction’, and ‘the irrelevance of the attitudes of those living in Tijuana is something that needs to be explained, not assumed, by a theory of law’.^111^

Looking at these matters through the lens of an official story suggests a different answer. If legal systems rest on shared commitments to structures and sources that are ‘essentially common’—if ‘There can be no “private” legal system, just as there can be no private language’^112^—then their recognitional communities ought to resemble those of other artificial normative systems, such as games or natural languages, which are likewise the common property of all who consult them. This suggests a view of the recognitional community as the community of people who jointly employ these normative systems as ‘essentially common’ standards of behaviour. Chess is defined by the chess players, those who recognise each other as playing the same game; law is defined by the law users, those who recognise each other as applying the same body of law. In slogan form, *the people whose shared acceptance matters are those who share acceptance*.

In other artificial normative systems, the members of the recognitional community in some sense recognise each other in the course of recognising their rules. Consider Joseph Raz’s example of someone who describes an object as a ‘table’, only to be corrected that it is actually a ‘drawing-board’.^113^ The correction is appropriate only in the context of an attempt to participate in a shared practice:

They meant to use ‘table’ in its so-called ordinary meaning. They had a view on what this meaning is, and they made a mistake. … Overhearing a conversation between others, I may realise that whereas I always thought that this object is a table in fact it is not, and my understanding of the rule for the use of ‘table’ … was mistaken.^114^

By contrast, overhearing a group of Italian speakers refer to the object as a ‘tavolo’ would not suggest any mistake on the English speaker’s part: the Italian speakers are obviously engaging in a separate practice with separate rules.

We have no settled view of the details of this mutual recognition. Arguably community members balance, in reflective equilibrium, their pre-existing notions of the rules’ content with their pre-existing notions of community membership. Thus, one might learn obscure rules of chess, such as taking pawns *en passant*, from those who already seem to be part of the chess-playing community; yet anyone playing the game *very* differently is probably playing a different game. Or a rule-governed practice might undergo a partial schism, such as that between British and American English, whose participants only partly regard each other as trying to follow the same rules. In any case, membership in a recognitional community seems likely to be a matter of degree—as seems appropriate for participation in ‘a complex, but normally concordant, practice’.^115^

To respond to Green’s Tijuana example, then, the recognitional community for California law might be similar in structure to that for American English. It might extend more or less to a broad swath of people, within California and without, who take its social rules more or less as shared standards of behaviour, and who seek to offer more or less the same official story of its law. Just as French grammarians can write influential guides to American English usage, insofar as these guides are taken as influential by the American-English-speaking community, ‘New Zealander[s] trained at Oxford’ might be important authorities on California law, insofar as the community of California law users take them to be so.^116^ But the recognitional practices of those living in Tijuana are not generally viewed by the community of California law users as part of their own, so the beliefs and practices of the former do not generally figure in the determination of California law. Even if more people claimed to be members of the community outside California than within it—just as more people claim to speak English outside England than within it—this might be evidence only of a geographic schism in the practice of California law; the extent to which one strand or another determines the law in the region known as California depends on the extent to which it is actually cited or invoked there.^117^ (Indeed, the existence of a distinct region known as California, with a distinct legal system of analytical interest, is something to be inferred from these various patterns of social behaviour rather than a predetermined first step in their analysis.) Recognitional communities are defined by the actual ways in which their members recognise one another; the content and the communities are determined together.

### D. Officials and Experts

Returning to Green’s concern about popular ignorance of the California Constitution, how might the members of recognitional communities recognise their shared rules (or even each other) if they generally know so little about them? One method might be to outsource their views to others. Citizens who are uncertain of their law need not look only to officials for guidance; they may also look to the views of experts, those whose understandings of a community’s rules are regarded by its members as good evidence of *their own* commitments and practices.

When employing social rules relating to language, etiquette or games, we often use what Hilary Putnam called the ‘division of linguistic labor’: we speak of elms and beeches as distinct trees, or of aluminium and molybdenum as distinct metals, without having any idea what distinguishes them, and trusting in expert botanists and metallurgists to know the difference.^118^ We might look up in written sources the etiquette of table setting (say, that forks go on the left), the ‘popular names’ of constellations or the rankings of uncommon poker hands; to look these things up is just to borrow, as evidence of our own customs, the views of others whom we regard as experts on the rules.^119^ Such rules exist, as intelligible features of social life, to the extent that there is sufficient social convergence on their content, on the identities of the experts who know about them or perhaps on some reflective-equilibrium combination of the two.

The same goes for rules of recognition. Jeremy Waldron points out that a society’s legal culture is rarely ‘confined exclusively to the corps of ruling officials’; the latter’s ‘relations with the general populace’ are ‘mediated’ by ‘specialist law-detectors (attorneys) who know the marks of law’, and whose knowledge ‘will be diffused beyond the profession in more or less amateurish ways’.^120^ Hart more than once includes experts in his corps of legal officials; he writes that citizens need not share a deep understanding of the law and its sources so long as one is held by ‘the officials *or the experts* of the system’, including ‘the lawyers whom the ordinary citizen consults when he wants to know what it is’.^121^ (And sometimes these experts call in experts—say, in complex cases of admiralty or tax law.^122^) The ‘marks of law’ Waldron describes are precisely the structures and sources that feature in the official story: those which legal actors publicly advance and defend, often by invoking their expertise.

Perhaps this view of the recognitional community is overly convenient for academics: the real work of recognition is done not by officials, but by expert law professors! But there is also a sense in which officials plainly do play a subservient role. It is coherent to argue, for example, that the US Supreme Court ‘astonished the ERISA bar’ by making ‘an elementary error in applying long-settled principles of trust law’.^123^ Such a claim is coherent only because the legal community regards an ‘invisible college’ of trust experts as actually knowing trust law, whereas the justices might be playing catch-up.^124^ That said, the officials may well be experts themselves, or they may have great causal influence over others’ views, reshaping the subject of the experts’ expertise. Or the officials may simply possess substantial rule-conferred authority within the system: lower courts still must follow higher ones, though the experts disagree on the merits. If officialdom diverges enough from the expert consensus, then this consensus may break down or change, just as a game may eventually change if the umpires insist on different rules. But which side will win the face-off is an empirical question, not a conceptual one.

What distinguishes this narrow class of experts from a narrow class of officials is the former’s essentially *public* nature. Not only do experts write for public consumption, but their expertise is a matter of degree, as determined by its reception. Sheltered academics count as experts only to the extent that others pay attention to their work (including others regarded by the public as experts, such as practitioners or other academics). And the experts’ role is not to settle the matter in isolation from the public, but to have their views incorporated by reference as part of the public’s own sense of its practices.

By contrast, the official world of the *Illuminati* hypothetical involves no such incorporation by reference, leaving it open to potential paradox. Consider the following scenario:

*Meta-Illuminati*: The Illuminati Order appears to most of its initiates entirely ordinary. The Order’s officials explain their decisions by citing the Order’s nefarious rules, and rank-and-file members implement these decisions under colour of US law. But a few canny members come to realise that the Order’s officials are all part of an even more secretive group (the Federalist Society), choosing outcomes according to the rules of the original US Constitution while purporting to justify them under the Order’s rules.

Should we say that the rules of the original US Constitution *are* the rules of the Illuminati? Or—as seems plain to us—have the Illuminati officials formed a conspiracy to *subvert* the Order’s rules?

The former answer seems to lead to contradiction. The garden-variety *Illuminati* hypothetical suggests that limits on congressional powers, say, are not rules of US law, while the rules of the Illuminati are; there is a systematic practice of US officials to ignore these limits whenever they believe an Illuminati rule requires otherwise (say, ‘do whatever hastens our control of world government’). But the *Meta-Illuminati* hypothetical suggests that the pursuit of world government *is not* among the Illuminati’s rules, while originalist limits on congressional powers are; there is a systematic practice of Order officials to ignore this pursuit whenever they believe the original Constitution requires otherwise. If the relevant social rules always and everywhere rest on willing acceptance by officials, then arguably the world-government rule both is and is not a rule of US law.^125^

By contrast, looking to the invisible college of trust experts produces no such paradox. Individual citizens might have all sorts of false beliefs about the law (dimming the prospects for any ‘deep popular constitutionalism’),^126^ but as a community they might nonetheless outsource their ‘official’ views to a community of expert lawyers. (They might even send their children to ‘law school’, hoping that it will confer on them some of this expertise.) On questions of trust law in particular, the community of expert lawyers might similarly outsource its views to a more specialised community of experts on trusts. A contradiction arises only when one *defines* the rules of each community to be those of some smaller subgroup, which might then be subject to further and unexpected subdivision; mere outsourcing involves no redefinition, simply a chain of incorporations by reference.

## 4. Efficacy

To the sceptic, all this might carry an air of unreality. A legal system, to exist in a particular time and place, presumably must have rules that are ‘generally obeyed’.^127^ In the *Illuminati* hypothetical, the valid rules according to the official story (say, originalist limits on congressional power) are *not* generally obeyed. Rather, they are generally *dis*obeyed, especially by those most responsible for enforcing them, whose alternative rules (such as wheat regulations) the public imagines to be entirely lawful. Clinging to the official story under these circumstances might seem naive or absurd; to suggest that Soviet law *under Stalin* protected free speech is arguably to make light of Stalinist repression or to abjure positivism, or both.^128^ Why insist that a society’s law depends on abstract forms of argument endorsed by experts when the ground-level rules that officials and the public know and follow rest on something else entirely?

The answer to this objection, like so much else in Hart’s theory, turns on the unique potential of secondary rules, which identify primary rules of behaviour in potentially unexpected ways. As a result, the efficacy that matters is the general *reliance* on these secondary rules, not actual compliance with the primary rules they actually identify. A given story can be the official story in a particular society even if it seems to call for remarkably different behaviour by officials.

On a Hartian account, legal systems are not just lists of on-the-ground prohibitions, but structured normative systems, with some rules held to be more fundamental than others. Secondary rules identify primary ones, sometimes in virtue of facts we do not yet know, so it is always possible to be surprised by what they entail. (‘If “whatever’s carved on this public monument is law”, we might discover new law whenever we dust off a previously overlooked carving.’^129^) That is why Hart rejects any ‘necessary connection between the validity of any particular rule and *its* efficacy’; legal rules might be obscure or violated in practice, and yet have unimpaired claims to validity, ‘exist[ing] as legal rules from the moment of their enactment before any occasion for their practice has arisen’.^130^ The efficacy condition is a condition for a normative system as a whole, not for any particular rule within that system: a society might have breathtaking levels of tax evasion and still have tax law.

Even in the *Illuminati* hypothetical, then, the orthodox legal system (though submerged) remains efficacious enough. Hart suggests that, for a legal system to exist, ‘the laws which are valid by the system’s tests of validity’ must be ‘obeyed by the bulk of the population’^131^—a test the orthodox legal system appears to fail. But in identifying a social norm, why is the degree of actual obedience the test, rather than the degree of obedience that is intended and perceived? The *Illuminati* hypothetical in no way resembles the cases of ‘Revolution’ or ‘Enemy occupation’ with which Hart is concerned,^132^ for the public still looks to the orthodox legal system for an account of its legal obligations, and many citizens think of themselves as law-abiding in its terms (though misled by officials as to what those terms are). The official story of orthodox US law in some sense plainly *obtains* there, in a way that (say) the official story of Roman law does not.

Insisting on actual rather than perceived obedience appears to share the sheeplike-society error discussed above. One cannot always demand that ordinary citizens employ a rule of recognition; sometimes the best evidence of a legal system’s existence is that private citizens habitually obey the law. But in ordinary legal systems, many citizens *do* generally subscribe to the official story, either directly or through trust in others’ expertise: they employ it as a standard of behaviour in identifying legal rules, think themselves in compliance with whatever law it generates and so on. One cannot infer from the extraordinary case the conclusion that, in ordinary cases, the citizens’ inadvertent failure to apply a social rule correctly somehow causes it no longer to be the rule of the group, or the legal rules it identifies no longer to be part of their legal system. A social rule can exist as an intelligible rule, crucially shaping its adherents’ thinking and conduct, even if no one manages to adhere to it successfully.^133^

Non-adherence can, of course, contribute to a rule’s eventual demise; and whether a given rule ‘is live or a dead letter comes in degrees’.^134^ If the Soviet official story effectively subordinated any free-speech protections to vague doctrines of state security, then it would be fair to describe these speech protections largely as dead letters. But the key feature of Soviet ‘telephone justice’ was that its results in politically sensitive cases were *not* easily explainable in orthodox Soviet legal terms. While their legal system was clearly efficacious overall, with a great deal of ordinary law and ordinary law breaking, Soviet judges nonetheless had to make an *effort* to dress up some of their decisions in legal vocabulary. This fact illustrates the gap between their law and their official practice: we can say that the Soviet Union had a *rule-of-law problem* because, far too often, the *law* was not what *ruled*.

In the same way, the real-world US legal system is plainly efficacious as a whole.^135^ This is so despite past official efforts to undermine the orthodox rules—as when Jim Crow courts sought to undermine the Fifteenth Amendment to the US Constitution.^136^ Their efforts found no home in the official story: the Amendment was not regularly enforced, but neither was it repealed, officially rendered a ‘dead letter’ or deprived of its status as valid law. All this was crucial to the ability of later generations to call for their law’s enforcement once again. The same may be true of other, similar changes in official practice that have left the official story unchanged.

## 5. Conclusion

Social rules exist in social groups; their content depends on what those who jointly accept them accept. This acceptance demands neither genuine belief nor even consistency in practice, but only the attitude of one who takes the rule as a shared guide to conduct and standard of behaviour. When participants lack direct knowledge of such a rule, the necessary consensus may be supplied by rough overlap among experts, and rough overlap among non-experts in judgments of expertise. To impose artificial limits on this universe of social rules, requiring genuine normative buy-in or an exclusive and well-defined official class, is a serious mistake; and if this is true of social rules in general, then it is also true of social rules that are used to identify the law. These views capture some of Hart’s most important insights about legal practice, and they give us good reasons not to disregard the official story of the law.

## Footnotes

1 Mikołaj Barczentewicz, ‘The Illuminati Problem and Rules of Recognition’ (2018) 38 OJLS 500, 504 (describing ‘telephone justice’).

2 Mark Jia, ‘Illiberal Law in American Courts’ (2020) 168 U Pa L Rev 1685, 1713–17.

3 Brian Leiter, ‘Legal Realism, Hard Positivism, and the Limits of Conceptual Analysis’ in Jules Coleman (ed), *Hart’s Postscript: Essays on the Postscript to* The Concept of Law (OUP 2001) 356.

4 See HLA Hart, *The Concept of Law* (3rd edn, OUP 2012) 100–1. Nothing here turns on the description of these norms as ‘rules’; we use ‘rules’ and ‘norms’ interchangeably, unless otherwise indicated.

5 See Stephen E Sachs, ‘The “Constitution in Exile” as a Problem for Legal Theory’ (2014) 89 Notre Dame L Rev 2253, 2265–8; Stephen E Sachs, ‘Originalism as a Theory of Legal Change’ (2015) 38 Harv J L & Pub Pol’y 817, 820, 870.

6 Barczentewicz (n 1) 503.

7 Richard A Posner, ‘Bork and Beethoven’ (1990) 42 Stan L Rev 1365, 1373.

8 See eg Barczentewicz (n 1) 503–4; William Baude and Stephen E Sachs, ‘Grounding Originalism’ (2019) 113 NWULR 1455, 1477–90. See also eg *Obergefell v Hodges* 576 US 644, 664 (2015) (‘The generations that wrote and ratified the Bill of Rights and the Fourteenth Amendment … entrusted to future generations a charter protecting the right of all persons to enjoy liberty as we learn its meaning’).

9 The Nomination of Elena Kagan to Be an Associate Justice of the Supreme Court of the United States: Hearing Before the S Comm on the Judiciary (2010) 111th Cong 62 (statement of Elena Kagan).

10 Hart, *Concept of Law* (n 4) 57. See also eg Lyle Denniston, ‘Argument Recap: An Uneasy Day for Presidential Power’ (*SCOTUSblog*, 13 January 2014) <https://perma.cc/VCK6-5UDM> accessed 24 March 2022 (describing ‘even some of the more liberal judges’ as ‘startle[d]’ at the open suggestion that ‘historical practice’ might trump ‘the words of the Constitution’); Jacob Gershman, ‘Posner Apologizes for Saying the Constitution is Useless’ (*Wall Street Journal Law Blog*, 1 July 2016) <https://wsj.com/articles/BL-LB-54096> accessed 24 March 2022.

11
*Wickard v Filburn* 317 US 111 (1942).

12 Richard H Fallon, Jr, ‘Constitutional Precedent Viewed Through the Lens of Hartian Positivist Jurisprudence’ (2008) 86 NC L Rev 1107, 1131.

13 See eg Mitchell N Berman, ‘Our Principled Constitution’ (2018) 166 U Pa L Rev 1325, 1347; Charles L Barzun, ‘Constructing Originalism or: Why Professors Baude and Sachs Should Learn to Stop Worrying and Love Ronald Dworkin’ (2019) 105 Va L Rev Online 128, 134–5.

14 See Richard Primus, ‘Is Theocracy Our Politics?’ (2016) 116 Colum L Rev Sidebar 44, 56–7. See also Baude and Sachs (n 8) 1486–7.

15 Paul Brest, ‘The Misconceived Quest for the Original Understanding’ (1980) 60 BU L Rev 204, 234.

16 Barczentewicz (n 1) 504.

17 David A Strauss, ‘Common Law Constitutional Interpretation’ (1996) 63 U Chi L Rev 877, 885.

18 Brian Leiter, ‘Constitutional Law, Moral Judgment, and the Supreme Court as Super-Legislature’ (2015) 66 Hastings LJ 1601, 1601.

19 Eric Posner, ‘Originalism Class 4: Brown’ (*Eric Posner*, 30 January 2014), <http://ericposner.com/originalism-class-4-brown/> accessed 24 March 2022.

20 Primus (n 14) 51.

21 ibid 56.

22 ibid. But see ibid 57 (preferring the motivated-reasoning explanation).

23 Kent Greenawalt, ‘Constitutional and Statutory Interpretation’ in Jules L Coleman, Kenneth Einar Himma and Scott J Shapiro (eds), *The Oxford Handbook of Jurisprudence and Philosophy of Law* (OUP 2004) 301 fn 36 (raising this possibility).

24 See Baude and Sachs, ‘Grounding Originalism’ (n 8) 1484–7, 1489–90.

25 Adapted from William Baude, ‘Is Originalism Our Law?’ (2015) 115 Colum L Rev 2349, 2388.

26 Barczentewicz (n 1) 508. See also ibid 519.

27 Mitchell N Berman, ‘Of Law and Other Artificial Normative Systems’ in David Plunkett, Scott J Shapiro and Kevin Toh (eds), *Dimensions of Normativity: New Essays on Metaethics and Jurisprudence* (OUP 2019) 143 fn 14. See also ibid 138–9 (describing the ‘Standard Positivist Picture’, which ‘bears a distinctly Hartian hue’).

28 ibid 159 (discussing the board game ‘Monopoly’).

29 Hart, *Concept of Law* (n 4) 116.

30 ibid 112; Thomas Adams, ‘The Efficacy Condition’ (2019) 25 LEG 225.

31 Hart, *Concept of Law* (n 4) 89–90.

32 cf ‘Professional Wrestling’ (*Wikipedia*, 8 February 2022) <https://en.wikipedia.org/w/index.php?title=Professional_wrestling&oldid=1070725775> accessed 24 March 2022 (‘Due to the staged nature of wrestling, these are not actual “rules” in the sense that they would be considered in similar articles about actual sports’).

33 Matthew D Adler, ‘Popular Constitutionalism and the Rule of Recognition: Whose Practices Ground U.S. Law?’ (2006) 100 NWULR 719, 732.

34 Barczentewicz (n 1) 512, 527.

35 Or perhaps we have two overlapping legal systems, each with its own participants, sources and demands; or we might have a fragmented legal system or none at all, as the necessary social consensus is gone; and so on.

36 Hart, *Concept of Law* (n 4) 98.

37 ibid 91.

38 ibid 56.

39 ibid 102.

40 ibid 57.

41 ibid 102.

42 See ibid 82–3, 85.

43 See ibid 98.

44 ibid 101.

45 ibid 116.

46 ibid 140.

47 See Michael A Wilkinson, ‘Is Law Morally Risky? Alienation, Acceptance and Hart’s Concept of Law’ (2010) 30 OJLS 441, 452, 454, 456 (arguing that Hart uses at least ‘two different versions of acceptance’, which he ‘fails properly to distinguish’—a ‘strong’ version that implies some genuine sense of ‘obligation’ and a ‘weak’ version that ‘simply means endorsement’).

48 Benjamin C Zipursky, ‘Legal Obligations and the Internal Aspect of Rules’ (2006) 75 Fordham L Rev 1229, 1241.

49 ibid 1238. See also Berman, ‘Artificial Normative Systems’ (n 27) 143 (discussing ‘ostensible normativity’).

50 Scott J Shapiro, ‘What is the Internal Point of View?’ (2006) 75 Fordham L Rev 1157, 1167.

51 Hart, *Concept of Law* (n 4) 57.

52 cf Berman ‘Artificial Normative Systems’ (n 27) 143–4 (describing ‘shnormative’ standards that produce ‘ostensible normativity’, ie standards that *would* be guides to conduct if the normative world were a certain way).

53 Adam Perry, ‘The Internal Aspect of Social Rules’ (2015) 35 OJLS 283, 288.

54 ibid 285. Perry emphasises belief independence to address an issue raised by GJ Warnock, *The Object of Morality* (Methuen 1977) 45–7 on how to differentiate social rules from other normative commitments (eg that one ought to take an umbrella if it might rain). For other potential answers, focusing on whether a rule belongs in a given *system* of rules, see Leslie Green, ‘Positivism and Conventionalism’ (1999) 12 CJLJ 35, 37; Jeffrey Kaplan, ‘Attitude and Social Rules, or Why It’s Okay to Slurp Your Soup’ (2021) 21 [28] Philosophers’ Imprint 1, 8–10.

55 Hart, *Concept of Law* (n 4) 57.

56 ibid 203. cf HLA Hart, *Essays on Bentham: Studies in Jurisprudence and Political Theory* (OUP 1982) 159 (arguing that a judge, when applying a legal rule, need neither ‘believe in [its] moral justification’ nor ‘pretend to believe in it’, and that, ‘without dereliction of his duty as a judge, he may have formed no view of the moral merits’), 265–6 (arguing similarly).

57 Shapiro (n 50) 1162.

58 Hart, *Concept of Law* (n 4) 57.

59 ibid 56.

60 ibid 91.

61 ibid 116.

62 ibid.

63 ibid.

64 We are indebted for this framing to an anonymous reviewer.

65 Barczentewicz (n 1) 510–11.

66 ibid. cf Neil MacCormick, *HLA Hart* (2d edn, Stanford UP 2008) 49 (discussing ‘willing acceptance’).

67 Barczentewicz (n 1) 511.

68 ibid 511, 511 fn 48.

69 Perry (n 53) 285. cf ibid 293 (analogising a disloyal judge to ‘an actor in a play’).

70 Hart, *Concept of Law* (n 4) 102.

71 See MacCormick (n 66) 45 (conceding that one may still have the proper attitude towards the rules of chess while cheating, so long as, ‘if caught, one acknowledges oneself to be in the wrong’). cf Gilbert Keith Chesterton, *The Man Who Was Thursday: A Nightmare* (Boni & Liveright 1908) 58 (‘Thieves respect property. They merely wish the property to become their property that they may more perfectly respect it’).

72 Joseph Raz, *The Authority of Law: Essays on Law and Morality* (OUP 1979) 153; MacCormick (n 66) 52.

73 See Paul Grice, *Studies in the Way of Words* (Harvard UP 1989) 92.

74 cf Don Fallis, ‘Lying as a Violation of Grice’s First Maxim of Quality’ (2012) 66 Dialectica 563, 569–72 (discussing statements made outside the usual norms of communication).

75 Perry (n 53) 291.

76 Robb Willer, Ko Kuwabara and Michael W Macy, ‘The False Enforcement of Unpopular Norms’ (2009) 115 American Journal of Sociology 451, 455, 458. See also ibid 458 (describing the fear of ‘being scorned as posers’ as ‘add[ing] to the peer pressure that leads others seeking social acceptance to join in’).

77 But see Minjae Kim, ‘Why Unpopular Practices May Persist as Ideal-Worker Norms: Signaling Commitment via Insincere Conformity’ 2, 20 <https://osf.io/nbe8a> accessed 19 August 2022 (noting that some groups enforce ‘visibly unpopular’ norms as a costly signal of conformity, rewarding compliance only ‘when it is *insincere*’, to the point that participants ‘try to hide that they actually enjoy conforming with the practice’).

78 See Nicholas Southwood and Lina Eriksson, ‘Norms and Conventions’ (2011) 14 Philosophical Explorations 195, 202–5 (arguing that norms can survive their universal—but non-public—violation).

79 Or consider another of Perry’s examples, that of ‘a spy or undercover agent who adopts the rules of a group to better infiltrate it’. Perry (n 53) 292. Suppose a government agent infiltrates a terrorist conspiracy, at great personal risk, only to discover that everyone there is *also* a government agent and that all the real terrorists left long ago (cf n 71). Before this discovery, the conspiracy was a real social group, with real social rules—a secret handshake, a standard ritual for executing informants, etc—some of which might have emerged only after the last real terrorist left. These rules may have been short-lived, and their acceptance a grave mistake; but one should not categorise them as anything but real social rules, when real people treated them as matters of life and death.

80 Barczentewicz (n 1) 512.

81 Perry (n 53) 291.

82 Adler (n 33) 733. See also Barczentewicz (n 1) 518; Kenneth Einar Himma, ‘Making Sense of Constitutional Disagreement: Legal Positivism, the Bill of Rights, and the Conventional Rule of Recognition in the United States’ (2003) 4 JL Soc’y 149, 154.

83 See Hart, *Concept of Law* (n 4) 114.

84 Leslie Green, ‘The Concept of Law Revisited’ (1996) 94 Mich L Rev 1687, 1700.

85 Hart, *Concept of Law* (n 4) 117.

86 ibid 116–17.

87 ibid 116.

88 Adler (n 33) 733. cf Green (n 84) 1702 (suggesting that, for Hart, ‘the only consensus necessary … is a consensus of elites’).

89 We are indebted for this example to John Harrison.

90 See Hart, *Concept of Law* (n 4) 115.

91 ibid 117.

92 ibid.

93 ibid 56.

94 ibid 116. See also Wilkinson (n 47) 450.

95 Wilkinson (n 47) 450. See also Richard Collins, ‘The Problematic Concept of the International Legal Official’ (2015) 6 TLT 608, 620 (discussing this problem).

96 Hart, *Concept of Law* (n 4) 144.

97 ibid 102.

98 ibid.

99 See ibid 142–4.

100 Himma (n 82) 154 fn 8.

101 Barczentewicz (n 1) 523.

102 See David Lefkowitz, ‘(Dis)solving the Chronological Paradox in Customary International Law: A Hartian Approach’ (2008) 21 CJLJ 129, 146 (similarly distinguishing the ‘ontological function’ of a rule of recognition from the ‘authoritative resolution’ such a rule might enable); Collins (n 95) 625–6 (discussing Lefkowitz). See also Grant Lamond, ‘The Rule of Recognition and the Foundations of a Legal System’ in Luís Duarte d’Almeida, James Edwards and Andrea Dolcetti (eds), *Reading HLA Hart’s* The Concept of Law (Hart Publishing 2013) 110 (‘Hart never explains what makes someone an “official” within a legal system, despite the fact that the distinction between officials and non-officials is invoked frequently through the text’).

103 See Collins (n 95) 619. See also Hart, *Concept of Law* (n 4) 142–4 (on ‘scorer’s discretion’).

104 MacCormick (n 66) 137. MacCormick questions whether this dependence on judges poses a vicious circularity, as references to ‘judges’ ‘presuppose a rule of adjudication’, which can be valid ‘only if it satisfies some criterion set in the rule of recognition’. ibid. Whether or not there is an escape from this circle—compare Collins (n 95) 616–19 (arguing that a dependence on officials is indeed circular) with Michael D Bayles, *Hart’s Legal Philosophy: An Examination* (Kluwer 1992) 81–3 (proposing distinct social rules of recognition and of adjudication, which separately identify the rules and the officials)—or an argument why the circle is not vicious—see eg Joseph Raz, *Ethics in the Public Domain: Essays in the Morality of Law and Politics* (OUP 1995) 296 fn 28; MacCormick (n 66) 140—the important point is that we need not enter the circle at all. See also Scott J Shapiro, *Legality* (Harvard UP 2011) 40 (discussing a related claim of chicken-and-egg circularity).

105 See Hart, *Concept of Law* (n 4) 94 (proposing that a rule of recognition ‘may be no more than that an authoritative list or text of the rules is … carved on some public monument’). See also Bayles (n 104) 82 (suggesting that ‘the Decalogue could constitute a simple rule of recognition without there being a system for adjudicating violations’). cf Barczentewicz (n 1) 520 (noting that officials are not the special addressees of the law, and that in any case a social rule is not determined solely by those whom it addresses, for a ‘rule that men ought to take their hats off in church may be a social rule of the whole society, including women and children’).

106 Hart, *Concept of Law* (n 4) 116.

107 ibid 40.

108 ibid 41. See also ibid 61 (noting that the ordinary citizen routinely ‘makes claims and exercises powers conferred by’ the law).

109 ibid 102.

110 ibid 110 (emphasis added). See also eg ibid 100 (the rule of recognition provides ‘both private persons and officials … with authoritative criteria’); ibid 101 (the rule is ‘manifest in the general practice’ of ‘officials or private persons’; ibid 107 (asking whether the use of the rule of recognition ‘is the practice of courts, legislatures, officials, or private citizens’).

111 Green (n 84) 1700–1. cf MacCormick (n 66) 49 (suggesting that the notion of group membership is crucial for ‘but not explained by Hart’).

112 Luka Burazin, ‘Legal Systems as Abstract Institutional Artifacts’ in Luka Burazin, Kenneth Einar Himma and Corrado Roversi (eds), *Law as an Artifact* (OUP 2018) 117.

113 Joseph Raz, *Between Authority and Interpretation: On the Theory of Law and Practical Reason* (OUP 2009) 64–5.

114 ibid 65.

115 Hart, *Concept of Law* (n 4) 110.

116 See Jeremy Waldron, ‘Minority Cultures and the Cosmopolitan Alternative’ (1992) 25 U Mich J L Reform 751, 756 (discussing ‘the common culture and civilization that makes it possible for a New Zealander trained at Oxford to write for a symposium in the *University of Michigan Journal of Law Reform*’).

117 Thus the linguistic rules of some alien civilisation numbering in the trillions, which learned a version of English from listening to our radio broadcasts, might properly be distinguished from Earth English unless recognised as part of a single tradition by earthlings.

118 Hilary Putnam, *Mind, Language, and Reality* (CUP 1975) 226–7. See also Stephen E Sachs, ‘Finding Law’ (2019) 107 CLR 527, 543.

119 See Sachs, ‘Finding Law’ (n 119) 535.

120 Jeremy Waldron, ‘All We Like Sheep’ (1999) 12 CJLJ 169, 180–1.

121 Hart, *Concept of Law* (n 4) 60 (emphasis added). See also ibid 61 (describing the official class as including ‘the legislators’, ‘the courts’ and ‘the experts when they guide the ordinary citizens by reference to the laws so made’). cf Barczentewicz (n 1) 516 (requiring officials to ‘hav[e] the capacity to guide action of non-officials’, a condition that experts appear to satisfy).

122 Sachs, ‘Finding Law’ (n 119) 543.

123 John H Langbein, ‘The Supreme Court Flunks Trusts’ (1990) 1990 Sup Ct Rev 207, 208.

124 See Collins (n 95) 628 (describing ‘the “invisible college” of international lawyers’—namely, ‘the diverse range of legal advisors, academics, negotiators, judges, and so on, who participate in the practice of international law’), citing Oscar Schachter, ‘The Invisible College of International Lawyers’ (1977) 72 NWULR 217. cf Barczentewicz (n 1) 520–1 (suggesting that many legal experts are not officials).

125 Perhaps one might contend that the Order’s rules are social rules, for which the recognitional community is not limited to officials, while the recognitional community for legal rules must be so limited. But that would require an explanation of why law merits such very different treatment from similarly structured normative systems.

126 See Adler (n 33) 726.

127 Hart, *Concept of Law* (n 4) 116.

128 Samantha Lomb, *Stalin’s Constitution: Soviet Participatory Politics and the Discussion of the 1936 Draft Constitution* (Routledge 2018) 135 (noting that early attempts to exercise speech rights under the new Soviet Constitution led to ‘mass arrests of “anti-Soviet” elements’).

129 Baude and Sachs, ‘Grounding Originalism’ (n 8) 1468. See generally Sachs, ‘Constitution in Exile’ (n 5).

130 Hart, *Concept of Law* (n 4) 103, 256.

131 ibid 114.

132 ibid 118.

133 See Southwood and Eriksson (n 78) 202–5.

134 Stefan Sciaraffa, ‘The Ineliminability of Hartian Social Rules’ (2011) 31 OJLS 603, 620.

135 See Brian Leiter, ‘Explaining Theoretical Disagreement’ (2009) 76 U Chi L Rev 1215, 1227 (describing ‘*massive* and *pervasive* agreement about the law throughout the system’).

136 See *Giles v Harris* 189 US 475 (1903).

## Notes

We are grateful for advice and comments to Thomas Adams, Matthew Adler, Hrafn Asgeirsson, Mikołaj Barczentewicz, Charles Barzun, Samuel Bray, Nathan Chapman, Ryan Doerfler, Kenneth Ehrenberg, John Goldberg, John Harrison, Mark Jia, Brian Leiter, Sandra Mayson, Ralf Michaels, Adam Perry, Eric Posner, Jeff Powell, Ketan Ramakrishnan, Richard Re, Frederick Schauer, Amanda Schwoerke, Lawrence Solum, Jeremy Telman and Adam Tucker; to participants in the Dartmouth College Law & Philosophy Workshop, the Duke University School of Law Faculty Workshop, the Oxford University Jurisprudence Discussion Group, the University of Chicago Public Law Workshop, the University of Georgia School of Law Faculty Workshop, the University of Virginia School of Law Faculty Workshop and the Yale Law School Legal Theory Workshop; and to Adam Griffin, Samuel Lewis and Scotty Schenck for excellent research assistance. Support is provided in part by a gift to Duke University School of Law from the Eugene T Bost, Jr, Research Professorship of The Cannon Charitable Trust No 3 and in part by the Alumni Faculty Fund and SNR Denton Fund at the University of Chicago Law School.

